# Standards of In-Patient Medical Seclusion Reviews

**DOI:** 10.1192/bjo.2024.400

**Published:** 2024-08-01

**Authors:** Mohammed Murtza, Taha Anjum

**Affiliations:** RCPsych, Leeds, United Kingdom

## Abstract

**Aims:**

Seclusion is a method used by mental health teams around the world to manage aggressive and disturbed behaviour in psychiatric patients in situations where there is immediate risk of harms to others.

A quality improvement project was carried out over two hospital in-patient sites containing 6 wards to see if seclusion reviews were completed safely and documented appropriately according to the guidelines set out by South West Yorkshire Mental Health Trust and the Royal College of Psychiatrists.

**Methods:**

The quality improvement project was carried out to first audit data to see if seclusion reviews were being done to the local guidelines and standards set by the Mental Health Legislation and Royal College of Psychiatrists. This was followed by training junior doctors and reauditing date to see if any improvements were observed.

A retrospective quality improvement project was conducted assessing medical seclusion reviews carried out by on-call junior doctors between November 2022 and January 2023. Data was initially collected retrospectively spanning over a period of 4 weeks over the month of November 2023 including the analysis of 30 seclusion reviews. These results were presented as an audit to doctors and managerial staff at the end of November. Post training seclusion review data was collected over a period of 4 weeks over January 2023.

**Results:**

An overall improvement in 7/9 domains. The biggest improvement (54% rise) was checking for side effects and EPSEs which was only documented 4/23 times in the first pre-training run. 18% improvements were also noted for assessing and documenting if the patient had any distress or pain, clinical appearance in terms of the cardiac domains such as perfusion and colour of the skin and also their level of orientation in place person and Glasgow Coma Score.

The only two domains in which an increase was not observed was to document if seclusion should continue and justification for why this is the case. These two domains were already at 100% and the System 1 seclusion review template prompts doctors to do this at the end of the review which is possibly one of the reasons it was done well both before and after the training.

**Conclusion:**

A great deal of interest and feedback was garnered and the idea was agreed that a further audit could be carried out after providing training for the current doctors and to gather post-training medical seclusion review data for comparison.

